# Supplementary material for: What is a meaningful life for persons with acquired neurological impairments? A scoping review protocol
Source: PLoS One. 2022 Jun 16;17(6):e0269125. doi: 10.1371/journal.pone.0269125 (PMC9202831; doi:10.1371/journal.pone.0269125)
Supplement: S1 Appendix — (DOCX) [file pone.0269125.s001.docx]

### **Appendix I: Search strategy**

## Search strategy for MEDLINE

Population

("Nervous System Diseases"[MeSH Terms] OR ("brain injur*"[Text Word] OR "spinal cord trauma*"[Text Word] OR "spinal cord injur*"[Text Word] OR "spinal cord transection*"[Text Word] OR "spinal cord lacer*"[Text Word] OR "traumatic myelopath*"[Text Word] OR "spinal cord contusion*"[Text Word] OR "stroke*"[Text Word] OR "apoplex*"[Text Word] OR "cerebrovascular accident*"[Text Word] OR "brain vascular accident*"[Text Word] OR "multiple sclerosis"[Text Word] OR "disseminated sclerosis"[Text Word] OR "brain laceration*"[Text Word] OR "parkinson*"[Text Word] OR "paralysis agitans"[Text Word] OR "amyotrophic lateral sclerosis"[Text Word] OR "lou gehrig disease*"[Text Word]))

AND

Content

("Sense of Coherence"[MeSH Terms] OR ("meaningful life"[Text Word] OR "meaning in life"[Text Word] OR "meaning of life"[Text Word] OR "life meaning*"[Text Word] OR "meaningful everyday life"[Text Word] OR "meaningful living"[Text Word]))

AND

Context

("Rehabilitation"[MeSH Terms] OR "rehabilitat*"[Text Word])

Combination of all

("Nervous System Diseases"[MeSH Terms] OR ("brain injur*"[Text Word] OR "spinal cord trauma*"[Text Word] OR "spinal cord injur*"[Text Word] OR "spinal cord transection*"[Text Word] OR "spinal cord lacer*"[Text Word] OR "traumatic myelopath*"[Text Word] OR "spinal cord contusion*"[Text Word] OR "stroke*"[Text Word] OR "apoplex*"[Text Word] OR "cerebrovascular accident*"[Text Word] OR "brain vascular accident*"[Text Word] OR "multiple sclerosis"[Text Word] OR "disseminated sclerosis"[Text Word] OR "brain laceration*"[Text Word] OR "parkinson*"[Text Word] OR "paralysis agitans"[Text Word] OR "amyotrophic lateral sclerosis"[Text Word] OR "lou gehrig disease*"[Text Word])) AND ("Rehabilitation"[MeSH Terms] OR "rehabilitat*"[Text Word]) AND ("Sense of Coherence"[MeSH Terms] OR ("meaningful life"[Text Word] OR "meaning in life"[Text Word] OR "meaning of life"[Text Word] OR "life meaning*"[Text Word] OR "meaningful everyday life"[Text Word] OR "meaningful living"[Text Word])) AND ("danish"[Language] OR "english"[Language] OR "norwegian"[Language] OR "swedish"[Language])
