# Supplementary material for: What is a meaningful life for persons with acquired neurological impairments? A scoping review protocol
Source: PLoS One. 2022 Jun 16;17(6):e0269125. doi: 10.1371/journal.pone.0269125 (PMC9202831; doi:10.1371/journal.pone.0269125)
Supplement: Attachment — Submitted filename: Rebuttal letter Plos One_December 2021.docx [file pone.0269125.s002.docx]

Dear academic editor Sander L. Hitzig and reviewers

Thank you very much for the opportunity to respond to the reviewers' comments and for the opportunity to re-submit the manuscript entitled " What is a meaningful life for persons with acquired neurological impairments? A scoping review protocol.

I would like to thank the reviewers very much for positive remarks and constructive comments.

Please find our responses to the comments below. We have also highlighted our changes in the track change document so you can easily see where we have made improvements according to your valued recommendations.

Best wishes,

Randi Steensgaard

| Editor: |  |
| --- | --- |
| More details regarding your intended methodology for your scoping review  - details on which scoping review framework  - your inclusion/exclusion criteria  - how adhere to the PRISMA-SCR | Joanna Briggs – we have added this  We have elaborated on this.  We have used the checklist in the document with track chances to illustrate how we plan to describe this |
| More background information on the types of neurological impairments that might be included | We have added text in the introduction (line 45-52) |
| A more nuanced discussion of the impact of these impairments on the person's health and wellbeing | We have added text in the introduction and discussion |
| Discussion on the larger impact of this review | A good and important point – we have added that in the discussion section (line 159-173) |
| Protocol should be reported in sufficient detail for another researcher to reproduce all experiments and analyses | We have described our steps more clearly |
| Reviewer 1 |  |
| - How is neurological conditions being defined? Is this for all age groups? | We wish to be open to all neurological conditions. In our search we searched for:  ("Nervous System Diseases"[MeSH Terms] OR ("brain injur*"[Text Word] OR "spinal cord trauma*"[Text Word] OR "spinal cord injur*"[Text Word] OR "spinal cord transection*"[Text Word] OR "spinal cord lacer*"[Text Word] OR "traumatic myelopath*"[Text Word] OR "spinal cord contusion*"[Text Word] OR "stroke*"[Text Word] OR "apoplex*"[Text Word] OR "cerebrovascular accident*"[Text Word] OR "brain vascular accident*"[Text Word] OR "multiple sclerosis"[Text Word] OR "disseminated sclerosis"[Text Word] OR "brain laceration*"[Text Word] OR "parkinson*"[Text Word] OR "paralysis agitans"[Text Word] OR "amyotrophic lateral sclerosis"[Text Word] OR "lou gehrig disease*"[Text Word]))  We did limit the search for persons older than 18 years. |
| - How many reviewers will partake on each step of the methodology? How will screening occur. | We have clarified this (line 131-133). We are at least two independent reviewers conducting each step of the scoping review. |
| - Can you better define "grey literature"? Specifically, what methods did you use to explore the grey literature and what inclusion/exclusion you used to initially map out all available "grey literature"? | We have defined grey literature (line 112-113) |
| -Can you better define what you mean by "meaningful life"? | The aim of the study is to describe what a meaningful life is from a patient. perspective. This requires us to stay open to whatever the persons characterise as meaningful. Therefore, we have not made any prior statements or definitions on this topic. |
| - What scoping review methodology are you following and how is the PRISMA-SCR being adhered to? | We follow the Joanna Briggs Institute methodology for scoping reviews and have added PRISMA-SCR.  Throughout the entire document the track changes now provide information on how the PRISMA-SCR is being adhered. |
| -The Keywords are clear but for indexing questions I suggest that the terms should be extracted from MeSH. | We absolutely agree that this is a good idea. Neurology and rehabilitation are MESH terms.  Scoping review is not a MESH term. However, we will keep this keyword as it is more correct than those suggested in the MESH database. The same goes for meaningful life. |
| -It is necessary to justify the choice of this time limit (or lack thereof) | We only present a timeline for the work and we are uncertain about what we need to elaborate on concerning time limit. |
| -It is necessary to justify the inclusion of all study designs (e.g., why are other review papers going to be included and how will they be synthesized) | We include all designs in order to gain the best possible illumination of the topic. Inclusion of reviews are a widely discussed issue. We are aware of the interpretation and will use the reference list for both critical evaluations of the statements and line search. |
| -Despite being a literature review there is ethical issues associated with this type of research that should be reported- what does this look like in terms of the "the Helsinki II Declaration and Ethical Guidelines for Nursing Research in the Nordic Countries" cited | Ethical considerations are described (line 175 – 178)  The fact that we do not include participants directly there are some ethical aspects we donot have to account for. However, according to the Ethical guidelines for Nursing research in Nordic countiries (<https://ssn-norden.dk/wp-content/uploads/2020/05/ssns_etiske_retningslinjer_0-003.pdf>)  the researcher has a duty to comply with the ethical guidelines laid down in the legislation, conventions or declarations which apply to all researchers. This duty covers all phases of the research process. The researcher should have good knowledge of methods and should possess the competence required by the project, both with regard to subject and to method. This means that the researcher must be qualified for and have experience in applying relevant methods for the collection and processing of data. Researchers who do not have research experience must work under the supervision of researchers with experience. The researcher shall ensure the safe storage of research materials. The researcher shall comply with prevailing national and international rules for authorship. Co-authorship entails that a substantial scientific contribution is made which comprises formulating the project or project-specific methods, or making an analysis and a critical interpretation of data, as well as participating in the formulation or critical examination of the manuscript. Further, it is the duty of the researcher to make all results publicly available and to publish them – including possible negative results.  We adhere to these aspects of the guideline. The same considerations are made about the Helsinki declaration where we adhere to the Scientific Requirements and Research Protocols.  (<https://www.wma.net/policies-post/wma-declaration-of-helsinki-ethical-principles-for-medical-research-involving-human-subjects/> ) |
| - What studies will be excluded and why? | We have added this (line 135-136, 140-141). |
| -The team present the categorization of the content of the articles from the bibliographic sample (i.e., through Covidence) - please clarify the methodological procedures and how this will be reported. Will results of the studies be included? How will themes be developed? | We have elaborated the methodological procedures (line 143-149) |
| Refer to the PRISMA-SCR item 13: Detail how evidence will be presented which may be in a narrative format, table, or visual representation, including a map or diagram | This is added. |
| - Please justify the use of the CASP in a scoping review (that typically does not assess methodological quality), why was this chosen? Will this apply to all study designs? | Thank you for being critical. Included studies are evaluated using a design specific quality assessment template i.e. the CASP checklist for qualitative studies (line 137-139). The quality assessment is needed, as we have no time limit for inclusion and the research question will predominantly be elucidated by qualitative studies where the quality has evolved over decades. |
| **Reviewer 2** |  |
| Introduction 1. Line 55 - Spell out acronyms the first time they appear (World Health Organization – WHO). | We have added this |
| 2. Good rationale for choosing to understand meaningful life in persons with neurological impairments – I would agree that participants thoughts about what they believe are meaningful to their quality of life are not often discussed. | Thank you for agreeing on this. |
| 3. The last sentence of the introduction could be more clear/concise. I would consider removing “what is known about the content of the ultimate goal of rehabilitation” since you are focusing on a meaningful life in the context of rehabilitation. | We have made the recommended change. |
| 1. What scoping review framework are you following for this review? This is a critical piece that is missing from this protocol. There are established frameworks that are widely used and are the gold standards for conducting scoping reviews. (Levac et al., 2010, - <https://pubmed.ncbi.nlm.nih.gov/20854677/> and the Joanna Briggs Institute | Joanna Briggs Institute methodology for scoping reviews. We have added this.  Throughout the entire document with track changes we have now provided information on how the PRISMA-SCR is being adhered to. |
| 2. It is a requirement of this journal that the appropriate PRISMA checklist is used for submission of scoping review protocols. Please mention that you are following the PRISMA guidelines and consider following their items for reporting your methods for this review. This will help ensure that your review is transparent and reproducible which is pivotal for this type of work. | Thank you for this comment. We have now added this. |
| 3. Eligibility Criteria – this needs to be further fleshed out.  a. Why are you focusing on those >18 years or older? Are you only including individuals who had their injury after that age? b. How are you conceptualizing meaningful life? What must articles discuss to be considered as “describing a meaningful life”? c. I am confused about line 89. Are you only including studies if they collected data in person? Does this exclude telephone surveys or online interviews? Please be more explicit by what you mean by this. | In and exclusion criteria has been fleshed out (line 97-113, 124-127)  a) Our aim is to understand the overall aim of individualized rehabilitation "a meaningful life" from a first-hand perspective. Therefore, we include autonomous persons suffering from injuries acquired as adults.  b) The aim of the study is to describe what a meaningful life is from a patient. perspective. This requires us to stay open to whatever the persons characterise as meaningful. Therefore, we have not made any prior statements or definitions on this topic.  Meaningful life must be expressed of the patients or cited in the included articles.  c) Sorry for that confusion. What we mean is that it should be a first-hand perspective. We hope this is clear. We include all methods – including telephone surveys if the questions are open-ended. |
| 4. Information sources – I am pleased to see that you are including grey literature as this is becoming more standardized for scoping reviews. However, not all grey literature is created equal (i.e., opinion pieces are not generally accepted). Please be more explicit with the grey literature you are choosing to use, how you will be searching for it and what sources you will use to search for the appropriate grey literature. | Grey literature including reports, expert opinions and editorials are also considered for inclusion in this scoping review (112-113). Initiating search using Google, Google Scholar and the ScoPus database which also contains references to conference proceedings and websites will be used. (not described in article due to word limit) |
| 5. Search Strategy – You say that an initial limited search will be done on 4 databases but the example search strategy you provided is for PubMed. Do you intend to search PubMed as one of your databases? PubMed is generally not recommended for scoping reviews because it is hard to replicate the search results, so I would suggest sticking with the initial databases you mentioned. Furthermore, you need to be more explicit about what databases you are using in your final search. | Medline (PubMed) was recommended by the Liberian in order to identify relevant text and keyword to develop the systematic search strategy.  Databases used (line 121-122). The search we have included in the article is just an example and we can include some of our additional searches as well. |
| 6. Data extraction process and critical appraisal: a. Will you be doing a pilot to ensure that the two independent reviewers understand the inclusion criteria? This is recommended in the frameworks (Levac, JBI) for conducting rigorous scoping reviews. If you are doing a pilot, will the remaining articles be split or will both reviewers review all the articles for inclusion. b. I appreciate that you are doing a quality assessment. I would suggest saying that you will be using the appropriate CASP checklist because there are multiple quality assessment templates that have been put out by them. | (a) We have elaborated the methodological procedures (line 129-141, 143-149)  (b) Thank you – please see line (137-138) |
| 7. Data analysis and presentation – the information you have presented here should actually be in the data extraction process. In the analysis section you should describe what techniques you will be using to summarize/collate the data, not what information you will be taking from the articles. | We agree and have elaborated in line 143-149 |
| Discussion 1. How could the information you obtain from the review be used by those working in rehabilitation? I think this is a major point missing from the discussion | We agree. Thanks for pointing this out. We have added a paragraph concerning this. |
